# Supplementary material for: Epidemiological trade‐off between intra‐ and interannual scales in the evolution of aggressiveness in a local plant pathogen population
Source: Evol Appl. 2018 Jan 4;11(5):768–80. doi: 10.1111/eva.12588 (PMC5979725; doi:10.1111/eva.12588)
Supplement: Supplementary file 1 [file EVA-11-768-s001.docx]

**Table S1** - *Zymoseptoria tritici* subpopulations (2 × 2 × 15 isolates) sampled in a wheat monoculture plot (Grignon, France).

| Ci-2009 | | |  | Cf-2010 | | |
| --- | --- | --- | --- | --- | --- | --- |
| Name | Code | Date of collection |  | Name | Code | Date of collection |
| INRA09-FS0729 | I01 | 24 November 2009 |  | INRA09-FS01000 INRA09-FS01002 INRA09-FS01003 INRA09-FS01006 INRA09-FS01008 INRA09-FS01013 INRA09-FS01015 INRA09-FS01018 INRA09-FS01019 INRA09-FS01021 INRA09-FS01022 INRA09-FS01023 INRA09-FS01024 INRA09-FS01025 INRA09-FS01026 | I16  I22  I23  I24  I25  I17  I26  I18  I27  I19  I28  I29  I20  I21  I30 | 12 July 2010 |
| INRA09-FS0731 INRA09-FS0732 | I06  I07 | 30 November 2009 |  |  |  |  |
| INRA09-FS0798 INRA09-FS0799 INRA09-FS0800 INRA09-FS0802 INRA09-FS0803 INRA09-FS0805 INRA09-FS0806 INRA09-FS0808 INRA09-FS0809 INRA09-FS0811 INRA09-FS0813 INRA09-FS0814 | I02  I08  I09  I03  I10  I11  I12  I04  I13  I14  I05  I15 | 8 December 2009 |  |  |  |  |

| Ai-2009 | | |  | Af-2015 | | |
| --- | --- | --- | --- | --- | --- | --- |
| Name | Code | Date of collection |  | Name | Code | Date of collection |
| INRA09-FS0402 INRA09-FS0406 INRA09-FS0410 INRA09-FS0411 INRA09-FS0414 INRA09-FS0417 INRA09-FS0420 INRA09-FS0421 INRA09-FS0423 INRA09-FS0425 INRA09-FS0439 INRA09-FS0434 INRA09-FS0438 INRA09-FS0444 INRA09-FS0452 | I31  I32  I33  I34  I35  I36  I37  I38  I39  I40  I41  I42  I43  I44  I45 | 9 October 2009 |  | INRA09-FS0265 INRA09-FS0266 INRA09-FS0267 INRA09-FS0268 INRA09-FS0269 INRA09-FS0270 INRA09-FS0271 INRA09-FS0278 | I46  I47  I48  I49  I50  I51  I52  I53 | 7 September 2015 |
|  |  |  |  | INRA09-FS0272 INRA09-FS0273 INRA09-FS0274 INRA09-FS0275 INRA09-FS0276 INRA09-FS0277 INRA09-FS0280 | I54  I55  I56  I57  I58  I59  I60 | 13 October 2015 |
